# Supplementary material for: Analysis of Mutational Status of IGHV, and Cytokine Polymorphisms as Prognostic Factors in Chronic Lymphocytic Leukemia: The Romanian Experience
Source: Int J Mol Sci. 2024 Feb 1;25(3):1799. doi: 10.3390/ijms25031799 (PMC10855205; doi:10.3390/ijms25031799)
Supplement: Supplementary file 1 [file ijms-25-01799-s001.zip › ijms-2783897-supplementary.pdf]

Supplementary Materials, Table S1. Clinical characteristics of CLL patients based on association of SHM with CNVs and/or somatic mutations, and variant alleles of the four studied SNPs

Variable	Mutated *IGHV* (%)	Unmutated *IGHV* (%)	p value	Unmutated *IGHV* with CNVs(%)	p value	Unmutated *IGHV* withsomatic	p value	Unmutated *IGHV* with somatic	p value	*IL-10* rs1800896(%)		p value	*IL-10* rs1800872(%)		p value	*TNF-α* rs361525(%)		p value	*TNF-α* rs1800750(%)		p value
										TT	TC+CC		GG	GT+TT		GG	GA		GG	GA	
**Age, y**																					
<40	1 (0.8)	1 (0.8)	0.93	0 (0.0)	0.52	0 (0.0)	0.65	0 (0.0)	0.32	1 (0.8)	1 (0.8)	0.55	2 (1.6)	1 (0.8)	0.29	2 (1.6)	0 (0.0)	0.90	2 (1.6)	0 (0.0)	-
40-65	13 (10.4)	13 (10.4)		17 (13.6)		7 (5.6)		3 (2.4)		25 (20.0)	39 (31.2)		28 (22.4)	36 (28.8)		63 (50.4)	1 (0.8)		64 (51.2)	0 (0.0)	
>65	8 (6.4)	10 (8.0)		23 (18.4)		3 (2.4)		7 (5.6)		18 (14.4)	41 (32.8)		33 (26.4)	25 (20.0)		56 (44.8)	2 (1.6)		58 (46.4)	1 (0.8)	
**Sex**																					
Men	14 (11.2)	14 (11.2)	0.56	15 (12.0)	**0.02**	6 (4.8)	0.93	6 (4.8)	0.93	30 (24.0)	48 (38.4)	0.32	37 (29.6)	41 (32.8)	0.89	77 (61.6)	1 (0.8)	0.12	78 (62.4)	0 (0.0)	0.19
Women	7 (5.6)	10 (8.0)		33 (26.4)		4 (3.2)		4 (3.2)		14 (11.2)	33 (26.4)		19 (15.2)	20 (16.0)		44 (35.2)	3 (2.4)		46 (36.8)	1 (0.8)	
**WBC, cells/µl**																					
<11000	7 (5.6)	8 (6.4)	0.73	2 (1.6)	**0.004**	1 (0.8)	0.18	1 (0.8)	0.18	6 (5.1)	13 (11.0)	0.46	10 (8.5)	9 (7.6)	0.86	18 (15.3)	1 (0.8)	0.85	19 (16.1)	0 (0.0)	0.60
≥11000	12 (9.6)	17 (13.6)		36 (28.8)		9 (7.2)		9 (7.2)		29 (24.5)	42 35.6)		39 (33.1)	32 (27.1)		68 (57.6)	3 (2.5)		70 (59.3)	1 (0.8)	
**PLT, cells/µl**																					
<150000	9 (7.2)	7 (5.6)	0.51	38 (30.4)	**<0.001**	3 (2.4)	0.98	3 (2.4)	0.98	16 (12.8)	31 (24.8)	0.51	24 (19.2)	23 (18.4)	0.97	46 (36.8)	1 (0.8)	0.81	47 (37.6)	0 (0.0)	0.41
≥150000	12 (9.6)	16 (12.8)		10 (8.0)		7 (5.6)		7 (5.6)		28 (22.4)	42 (33.6)		36 (28.8)	34 (27.2)		68 (54.4)	2 (1.6)		69 (55.2)	1 (0.8)	
**Hemoglobin, g/dl**																					
<13	11 (8.8)	20 (16.0)	**0.03**	14 (11.2)	**<0.001**	7 (5.6)	0.25	8 (6.4)	0.61	29 (23.2)	51 (40.8)	0.66	38 (30.4)	42 (33.6)	0.23	78 (62.4)	2 (1.6)	0.95	79 (63.2)	1 (0.8)	0.15

≥13	8 (6.4)	3 (2.4)		34 (70.8)		3 (2.4)		2 (1.6)		15 (12.0)	22 (17.6)		22 (17.6)	15 (12.0)		36 (28.8)	1 (0.8)		37 (29.6)	0 (0.0)	
**LDH, IU/l**																					
<480	17 (13.6)	19 (15.2)	0.53	34 (27.2)	0.22	9 (7.2)	0.65	9 (7.2)	0.65	36 (28.8)	59 (47.2)	0.89	51 (40.8)	44 (35.2)	0.28	92 (73.6)	3 (2.4)	0.39	94 (75.2)	1 (0.8)	0.63
≥480	2 (1.6)	4 (3.2)		14 (11.2)		1 (0.8)		1 (0.8)		8 (6.4)	14 (11.2)		9 (7.2)	13 (10.4)		22 (17.6)	0 (0.0)		22 (17.6)	0 (0.0)	
**LYMPH, %**																					
<25	2 (1.6)	1 (0.8)		1 (0.8)		0 (0.0)		0 (0.0)		0 (0.0)	5 (4.0)	0.48	3 (2.4)	2 (1.6)	0.94	5 (4.0)	0 (0.0)	0.42	5 (4.0)	0 (0.0)	-
25-40*	3 (2.4)	0 (0.0)	0.27	0 (0.0)	0.59	0 (0.0)	0.50	0 (0.0)	0.50	3 (2.4)	3 (2.4)		3 (2.4)	3 (2.5)		5 (4.0)	1 (0.8)		5 (4.0)	1 (0.8)	
≥40	14 (11.2)	22 (17.6)		47 (37.6)		10 (8.0)		10 (8.0)		35 (28.0)	61 (48.8)		51 (40.8)	45 (36.0)		94 (75.2)	2 (1.6)		96 (76.8)	0 (0.0)	

Data are presented as numbers (percentage); p values were obtained by the χ^2test; *Normal values of the laboratory.

Supplementary Materials, Table S2. Associations of genetic risk factors in CLL patients.

**No. of patients**	**Unmutated *IGHV***	***IL-10* rs1800896 variant allele C**	***IL-10* rs1800872 variant allele T**	***TNF-* α rs361525 variant allele A**	***TNF-* α rs1800750 variant allele A**	**Chromosomal aberrations**	**Somatic mutations**
24	yes	yes	no	no	no	yes	no
15	yes	yes	no	no	no	no	no
14	yes	no	yes	no	no	no	no
12	yes	yes	yes	no	no	no	no
11	yes	no	yes	no	no	yes	no
10	yes	yes	yes	no	no	yes	no
5	yes	no	no	no	no	no	no
5	yes	yes	yes	no	no	yes	yes
5	yes	no	no	no	no	yes	no
4	yes	yes	no	no	no	no	yes
3	yes	yes	yes	no	no	no	yes
3	no	yes	no	no	no	no	yes
2	yes	yes	no	no	no	yes	yes
2	yes	no	yes	no	no	no	yes
1	no	yes	yes	no	no	no	yes
1	no	no	no	no	no	no	yes
1	no	no	yes	no	no	yes	yes
1	yes	yes	no	yes	no	yes	yes
1	yes	no	yes	no	no	yes	yes
1	yes	no	no	no	no	yes	yes
1	yes	no	no	no	no	no	yes
1	yes	yes	yes	yes	no	no	no
1	yes	yes	no	yes	no	yes	no
1	yes	yes	no	yes	yes	no	no

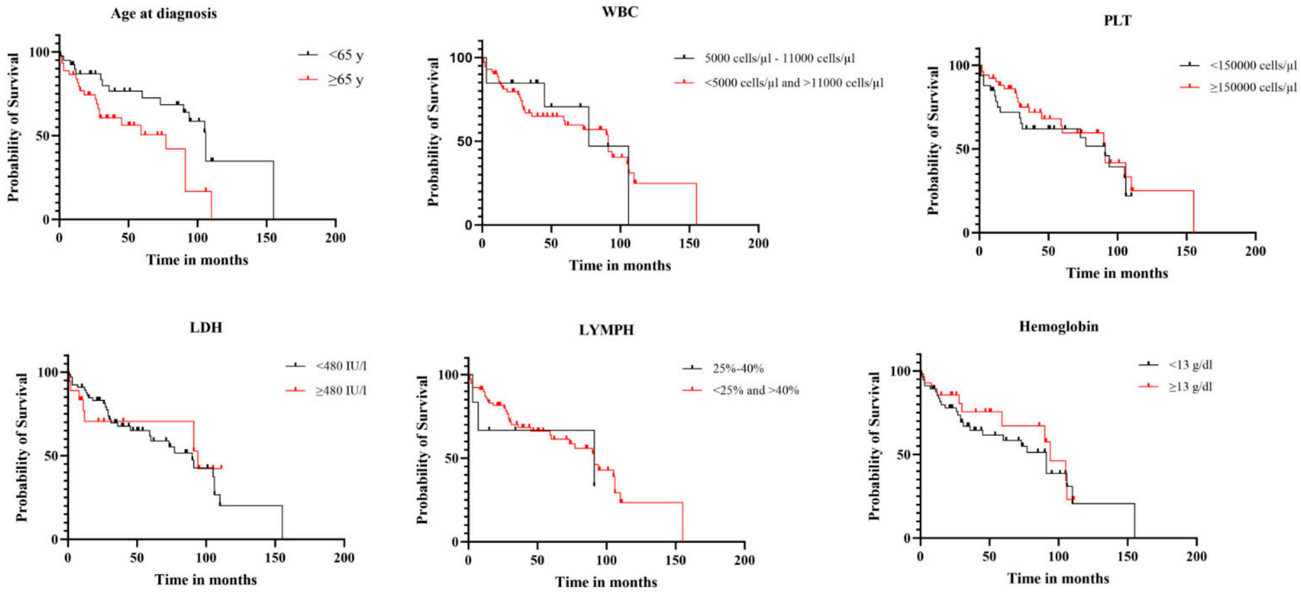

Supplementary Materials, Figure S1. Kaplan Meier curves for clinical characteristics of CLL patients
